# Evolution of Key Factors Influencing Performance Across Phases in Junior Short Sprints

**DOI:** 10.3390/sports12120321

**Published:** 2024-11-27

**Authors:** Kyosuke Oku, Yoshihiro Kai, Hitoshi Koda, Megumi Gonno, Maki Tanaka, Tomoyuki Matsui, Yuya Watanabe, Toru Morihara, Noriyuki Kida

**Affiliations:** 1 Faculty of Arts and Sciences, Kyoto Institute of Technology, Hashikami-cho, Matsugasaki, Sakyo-ku, Kyoto 606-8585, Japan; kyosuke.oku@gmail.com; 2 Department of Physical Therapy, Faculty of Health Sciences, Kyoto Tachibana University, 34 Yama-da-cho, Oyake, Yamashina-ku, Kyoto 607-8175, Japan; kai-y@tachibana-u.ac.jp; 3 Department of Rehabilitation Sciences, Faculty of Allied Health Sciences, Kansai University of Welfare Sciences, Asahigaoka 3-11-1, Kashiwara-shi 582-0026, Japan; h-koda@tamateyama.ac.jp; 4 Department of Childhood Education, Faculty of Childhood Education, Nagoya Women’s University, 3-40 Shioji-cho, Mizuho-ku, Nagoya-shi 467-8610, Japan; gonno@nagoya-wu.ac.jp; 5 Department of Childhood Education, Faculty of Human Development and Education, Kyoto Tachibana University, 34 Yamada-cho, Oyake, Yamashina-ku, Kyoto 607-8175, Japan; tanaka-mak@tachibana-u.ac.jp; 6 Marutamachi Rehabilitation Clinic, 12 Nishinokyo Kurumazakacho Nakagyo-ku, Kyoto 604-8405, Japan; matsui.tomoyuki.sports.reha@gmail.com (T.M.); toru4271@koto.kpu-m.ac.jp (T.M.); 7 Department of Sport Study, Faculty of Sport Study, Biwako Seikei Sport College, 1204 Kitahira, Otsu-shi 520-0503, Japan; watanabe-yuy@bss.ac.jp

**Keywords:** short sprint, across sections, body composition, flexibility, muscle strength, physical fitness test, junior athletes

## Abstract

Sprint performance plays a crucial role in various sports. Short sprints vary depending on the size of the court/playing field and on competitive characteristics, but are common in many sports. Although the relationship between age and muscle strength has been explored in short sprints, there is limited understanding of how various physical factors interact, particularly concerning differences in the acceleration phase. This study examined the relationship between sprint times at 0–2.5 m, 2.5–5 m, and 5–10 m intervals and various factors (body composition, flexibility, muscle strength, physical fitness) in junior athletes (13 boys; 13 girls; average age 11.37 ± 1.30 years; 7 in badminton, 8 in fencing, 5 in rowing, and 6 in climbing). The sprint time was measured using four timing lights at 0 m (start point), 2.5 m, 5 m, and 10 m (finish point). The results indicated that sprint times increased with age, and is correlated with muscle strength and flexibility. A partial correlation analysis showed that faster times in the 0–2.5 m interval were associated with higher hip flexibility (right: *r* = −0.42, *p* = 0.035; left: *r* = −0.60, *p* = 0.001); in the 2.5–5 m interval, faster times were associated with higher core flexibility (right: *r* = −0.34, *p* = 0.091; left: *r* = −0.40, *p* = 0.046); and in the 5–10 m interval, a relationship with standing long jump performance was confirmed (*r* = −0.56, *p* = 0.003). Furthermore, a lower fat-free body weight translated to higher performance (0–2.5 m: *r* = 0.40, *p* = 0.047; 2.5 m: *r* = 0.37, *p* = 0.071; 5–10 m: *r* = 0.55, *p* = 0.004). In the acceleration phase of 10 m, flexibility immediately after the start and the subsequent horizontal propulsive force are important factors that are strongly related to performance change in each interval. These results emphasize that even over a short distance such as 10 m, the factors influencing performance can change significantly. This highlights the importance of overall flexibility, propulsive power and body fat regulation in junior short sprinters, as well as the need for daily training carefully tailored to the specific sprint distances required in each sport.

## 1. Introduction

In various sports, it is necessary to demonstrate maximum performance within a limited space and time. Short sprints are considered crucial, particularly in sports involving locomotion. In tennis, sprints of less than 20 m have been highlighted as important [1], and predicting the time for a 5 m sprint can be performed based on four factors: age, height, lower-limb explosive strength (LLES), and skill level [2]. The relationship between performance and short sprints has also been reported in other sports such as handball, where differences in 30 m sprint times are based on skill level [3], and in hockey, where short sprints can serve as predictive factors for skill level [4,5]. Short sprints are closely related to performance in various sports and increasing the speed of short-distance movements leads to improved performance.

During short sprints, the acceleration phase immediately after the start of the sprint is crucial. The required sprint distance varies depending on the sport; however, the acceleration phase immediately after the start is common in all sports. Previous studies have shown differences in times of 2.5 m and 5 m based on the stepping technique at the start [6], and starting with one foot down rather than setting both feet parallel at the start has been confirmed to result in faster times at 5 m and 10 m [7]. Thus, the times for the initial 2.5 m, 5 m, and 10 m have been well studied, highlighting the importance of performance at these distances. Additionally, differences in the 5 m time are already evident at the 2.5 m point [6], suggesting that an analysis across sections of 0–2.5 m, 2.5–5 m, and 5–10 m separately may reveal important factors in each interval.

Short sprints are related to physical characteristics, such as age and body weight. A study examining 30 m sprints in the 11–15-years-old age group reported that as age increased, maximum speed, stride length, and ground contact time increased [8]. Regarding physical factors, body weight has a negative impact on maximum speed in 30 m sprints [9]. In tennis, body mass index (BMI) has been reported to affect athletic performance [10]. Therefore, considering the changes in body composition with age and growth, it is important to examine the key factors in short sprints.

Physical fitness and muscle strength are important factors for short sprints. Previous studies have shown a correlation between peak velocity in vertical jumps and 10 m sprint times [11,12], with vertical jumps also correlating with sprint times at 10, 30, and 60 m [13]. In a previous study, when participants were divided into two groups based on back squat scores, no significant time differences were observed in muscle strength at 5 m, but individuals with stronger lower-limb muscles were faster at 10 m and 20 m [14]. There have also been reports of a positive correlation between half-back squats and speed at the 5 m mark [15]. In terms of muscle volume in the lower limbs, there is no relationship between the semitendinosus and speed in the 0–10 m interval, but a positive relationship has been reported for speed in the 50–60 m interval [16]. Thus, the numerical values of lower-limb muscle strength and physical fitness tests are important in explaining short sprint performance. This study focused on lower-limb muscle strength and physical fitness tests to examine short sprints.

Maintaining physical flexibility is also important in short sprints. Previous studies have reported that the higher the flexibility of the hamstrings, the faster the times for 5 m, 10 m, and 20 m sprints [17]. In this study, we examined the flexibility of the lower limbs and that of the upper body. This facilitated a detailed investigation of the functionality of short sprints involving the entire body.

Our study aimed to elucidate the evolving key factors that strongly influence sprint performance in each interval—0–2.5 m, 2.5–5 m, and 5–10 m—during the junior years, a stage marked by significant physical development. This novel approach examines how performance factors shift across intervals, building on previous research that has highlighted how differences in 5 m times arise as early as the 2.5 m mark [6]. To clarify the factors essential for maximizing performance at each interval, it is crucial to isolate the influence of previous sections. By doing so, we can more accurately identify the specific elements required to enhance performance within each distinct section. Furthermore, focusing on the junior years—a period of significant age-related physical development—and comprehensively examining the relationship with body composition, muscle strength, flexibility, and motor skills adds further significance to this study. Our hypothesis posits that these key factors vary with each interval, reflecting the progression from a stationary to a running state, where different aspects are needed to maximize performance. Given the role of short sprints in various sports and the emphasis on start performance, this study also holds value in both coaching and rehabilitation, where understanding sprint performance across sections is crucial.

## 2. Method

### 2.1. Study Design

This study utilized a cross-sectional design to examine the factors influencing sprint performance in junior athletes aged 9–14 years. Participants regularly engaged in sports, and their sprint performance was evaluated over three short intervals within a 10-m sprint (0–2.5 m, 2.5–5 m, and 5–10 m). Physical measurements—including body composition, flexibility, muscle strength, and physical fitness tests—were taken, and their relationships with sprint times at each interval were analyzed. All participants completed all measurements within a single day, and the order of measurements was randomized for each participant. This design aimed to clarify how different physical characteristics correlate with sprint performance over a specific interval of the acceleration phase.

### 2.2. Participants

This study included 30 boys and girls who regularly participated in competitive sports, and the analysis was conducted on the 26 participants who had no missing values in the measurements (13 boys, 13 girls, average age 11.37 ± 1.30 years). With an effect size at the medium level of 0.3, a significance level of 0.05 and a sample size of 26, the power calculation using G*Power (3.1.9 for Mac) was 0.32. The participants specialized in the following disciplines: 7 in badminton, 8 in fencing, 5 in rowing, and 6 in climbing, with each engaging in specialized training sessions approximately twice a week. The training age of the participants was 22.23 ± 15.45 months. In this study, we focused on general sprint performance that is not specific to a particular sport, so we targeted elite junior athletes who do not use sprinting frequently in their sport. These athletes are taught sprinting form as part of a common program 2–3 times a year, but they do not train sprinting on a daily basis. Before the experiment, the purpose and procedure were explained to the participants and written informed consent was obtained. This study was approved by the Ethics Committee of the Kyoto Institute of Technology and conducted in accordance with the Declaration of Helsinki.

### 2.3. Short Sprint

**Figure 1:**
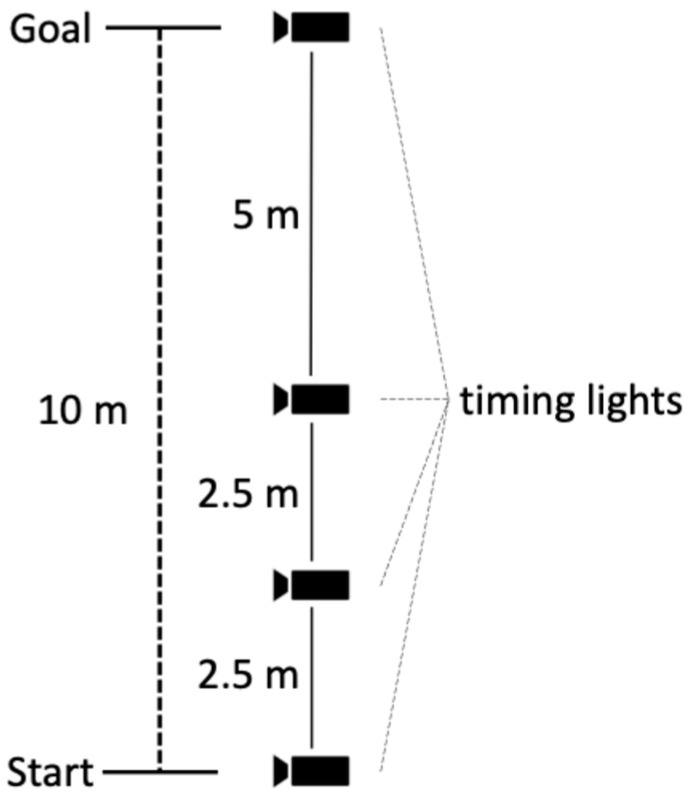
Overview diagram of short sprint measurement.

An overview of the measurement environment is presented in Figure 1. All measurements were conducted indoors with the participants using indoor shoes they brought with them. Short sprints were measured using a phototube (Dashr-Blue) placed at four positions: 0 m (start point), 2.5 m, 5 m, and 10 m (finish line). The height of the phototube was set at 80 cm. Participants were asked to stand still at a position where the tip of one foot touched the starting line and then to start running from a convenient position. The starting time was set at the discretion of the participants. Each participant performed the trial twice, and the best performance was selected for analysis. Passage times at 0–2.5 m, 2.5–5 m, and 5–10 m were calculated for analysis.

### 2.4. Body Composition

Body composition was measured using the InBody 570 (InBody Japan, Inc., Tokyo, Japan) for body weight, body fat mass, and fat-free mass.

### 2.5. Flexibility

The measurement of joint range of motion was conducted in accordance with methods outlined in previous studies [18]. Trunk rotation was performed with the pelvis fixed in a seated position. The angles between the lines connecting the left and right posterior superior iliac spines and the left and right acromion were measured. The hip flexion angle was measured with the pelvis fixed in a supine position, with the knee joint flexed naturally, and the angle between a line parallel to the trunk and the line connecting the femur (center of the greater trochanter and the lateral epicondyle of the femur) was measured. Measurements were conducted by groups of four physical therapists, with one therapist fixing the subject’s body to prevent compensatory movements, one therapist moving the subject’s body, one therapist measuring the angles and distances, and one therapist recording the data. Angles were measured in one-degree increments using a University of Tokyo-style goniometer (SAKAI Medical Co., Ltd., Tokyo, Japan).

### 2.6. Muscle Strength

In the muscle strength measurements, knee extension and flexion strengths were measured. Knee extension and flexion strength were measured using Cybex Norm (CSMi, USA, Inc., Stoughton, MA, USA) in a seated position by measuring isometric and isokinetic strength during extension and flexion movements. Isokinetic strength measurements were performed at 300°/s, and all participants underwent measurements on their right leg. Based on reports that there is no significant difference in muscle strength between the right and left foot [19,20,21] and reports that although asymmetry is seen between the two feet, a high correlation is confirmed [22], in this study, only the right foot was measured for both isometric and isokinetic tests.

### 2.7. Physical Fitness Test

Physical fitness tests included sit-and-reach, repeated side-steps, standing long jumps, sit-ups, and whole-body reaction times. Each participant was assigned an assistant to ensure safety during the fitness tests and other measurements. For the sit-and-reach test, participants sat on the ground with their backs against a wall, their legs fully extended, and were instructed to reach forward. The change in values from the initial arm extension to the post-reach position was recorded, and the best of two attempts was used [23,24]. For the repeated side-step, three lines were set 1 m apart, and participants were asked to side-step back and forth for 20 s. The number of times both feet crossed each line was counted and the best result of the two attempts was used [25,26]. In the standing long jump, the participants slightly widened their feet, aligned their toes with the starting line, and jumped forward simultaneously with both feet. The distance from the starting line to the back of the heels was measured, and the best result from two attempts was used [27,28,29]. For the sit-ups, the participants crossed their arms over their chests, kept their knees bent at a 90° angle, and had their feet secured by an assistant. The number of times the elbows touched both thighs within a 30 s period was measured. Each participant was given only one attempt [30,31]. Whole-body reaction time was measured by standing on a mat sensor and reacting as quickly as possible to visual stimuli placed at eye level by jumping slightly. The time from visual stimulus presentation to the feet leaving the mat switch was recorded [32].

### 2.8. Statistical Analysis

In order to clarify the important factors for sprint performance, we examined the correlation between sprint times for each section (0–2.5 m, 2.5–5 m, 5–10 m) and body composition, flexibility, muscle strength, and physical fitness tests. As the body undergoes significant age-related changes during the junior years, we also conducted a partial correlation analysis using age in month as a control variable. In this analysis, we accounted for the relative age effect, where even within the same school year, individuals with older birth months tend to have faster physical development, by using age in month as a control variable. All statistical analyses were performed using SPSS version 27 (IBM Japan, Ltd., Tokyo, Japan).

## 3. Result

**Figure 2:**
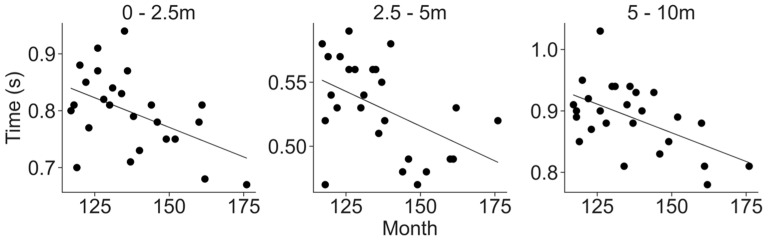
Relationship between age in month and sprint time in each interval.

The relationship between age in months and time in the 0–2.5 m, 2.5–5 m, and 5–10 m intervals is shown in Figure 2. Negative correlations were found between age in month and time in all intervals, indicating that sprint times in each interval decreased with an increase in age (0–2.5 m: *r* = −0.49, *p* = 0.01; 2.5–5 m: r = −0.47, *p* = 0.02; 5–10 m: r = −0.54, *p* = 0.004).

We conducted a simple correlation analysis between sprint times in the 0–2.5 m, 2.5–5 m, and 5–10 m intervals and various variables such as body composition, flexibility, muscle strength, and physical fitness tests (Table 1). In the 0–2.5 m interval, significant correlations were found with sprint time in the 5–10 m interval (*r* = 0.64, *p* = 3.81 × 10^−4^), isometric knee flexion (*r* = −0.48, *p* = 0.014), and standing long jump (*r* = −0.59, *p* = 1.35 × 10^−3^). In the 2.5–5 m interval, correlations were found with right–left trunk rotation (right: *r* = −0.47, *p* = 0.017; left: *r* = −0.53, *p* = 0.006), isometric knee extension (*r* = −0.45, *p* = 0.023), and standing long jump (*r* = −0.41, *p* = 0.038). In the 5–10 m interval, significant correlations were found between sprint time (*r* = 0.64, *p* = 3.81 × 10^−4^), fat-free mass (*r* = −0.42, *p* = 0.032), body fat mass (*r* = 0.45, *p* = 0.022), height (*r* = −0.45, *p* = 0.022), isometric knee flexion (*r* = −0.60, *p* = 0.001), isotonic knee flexion (*r* = −0.44, *p* = 0.023), repeated side-step (*r* = −0.39, *p* = 0.049), standing long jump (*r* = −0.72, *p* = 3.42 × 10^−5^), and whole-body reaction time (*r* = 0.40, *p* = 0.044).

The simple correlation analysis performed in this study revealed a relationship between age in months and performance in all three intervals. Therefore, we examined the correlation between age in months and other factors (Table 2). Consequently, a significant correlation was confirmed with all factors except for body fat mass (range of absolute value, *r* = 0.39 to 0.79; *p* < 0.05). Therefore, age (in months) may have a potential influence on identifying factors that are strongly related to sprint time in each interval.

We conducted partial correlation analyses between each time interval (0–2.5 m, 2.5–5 m, 5–10 m) and various variables using confirmed age in months with relationships with many variables as a control variable (Table 3). As a result, in the 0–2.5 m interval, a tendency was observed for faster times with higher hip flexibility (right: *r* = −0.42, *p* = 0.035; left: *r* = −0.60, *p* = 0.001). In the 2.5–5 m interval, a tendency for faster times with higher trunk flexibility was observed (right: *r* = −0.34, *p* = 0.091; left: *r* = −0.40, *p* = 0.046). In the 5–10 m interval, a tendency for faster times with better standing long jump records was observed (*r* = −0.56, *p* = 0.003). Furthermore, the previously non-significant body fat mass in the simple correlation analysis was found to have partial correlations with all interval times (0–2.5 m: *r* = 0.40, *p* = 0.047; 2.5 m: *r* = 0.37, *p* = 0.071; 5–10 m: *r* = 0.55, *p* = 0.004).

## 4. Discussion

This study aimed to identify the key factors that contribute to short sprint performance among junior students, with a particular focus on how these factors change over different intervals. The results of this study showed that in the simple correlation analysis, factors related to the muscular system, such as knee flexion and extension, were confirmed at many intervals. However, in the partial correlation analysis considering the influence of age in months, flexibility factors were strongly related in the first half distance of 10 m, specifically 0–2.5 m and 2.5–5 m. When examining each interval, it was confirmed that in the 0–2.5 m distance, high flexibility of the hip joint, which did not show a relationship in the simple correlation analysis, led to faster times in that distance (pseudo-uncorrelation). In the 2.5–5 m distance, it was revealed that even when considering the influence of age in months, the flexibility of the trunk, which showed a relationship in the simple correlation analysis, was related. Thus, when considering the influence of age in month, it became clear that especially in the first half segments of 10 m, 0–2.5 m, and 2.5–5 m, sprint performance improves with higher flexibility. In the 5–10 m distance, the standing long jump, which showed a relationship in both simple and partial correlation analyses, suggests that the ability to generate a horizontal propulsive force is important. Therefore, in the junior period, when considering the influence of age in months, it became evident that factors with strong relationships change even at short distances such as 10 m, with hip joint flexibility changing at 0–2.5 m, trunk flexibility at 2.5–5 m, and horizontal propulsive force at 5–10 m.

In terms of physical elements, body fat mass at all intervals negatively impacted sprint time, even during the junior period. This finding is highly consistent with and reinforces previous studies reporting that heavier weights are associated with slower sprint times [9,33]. As a longer stride is a critical factor in achieving greater acceleration, an increase in body fat mass may influence movement patterns and ultimately result in slower times. Taken together, the results of both previous studies and the present study clearly show that an increase in body fat mass contributes to an increase in weight, which negatively affects short sprint performance, even in juniors.

In the initial sections of 0–2.5 m and 2.5–5 m, faster times were associated with greater flexibility, enabling longer strides. In this study, it became evident that higher flexibility in the hip joints leads to faster times in the 0–2.5 m section, while higher flexibility in the trunk results in faster times in the 2.5–5 m section, highlighting the importance of body flexibility immediately after the start. Previous studies have reported that a greater step width in the 0–5 m section leads to higher acceleration [34]. Furthermore, a larger step length in the first step after the start results in greater propulsive force, suggesting immediate performance improvement [35]. Taken together, these results suggest that increasing the step length is crucial for achieving high acceleration, with hip and trunk flexibility enabling such a step length.

In particular, hip flexibility is considered specific to the junior period. Previous studies examining power output by joints at the start have reported that hip power output plays a significant role in young athletes, whereas knee power output accounts for a larger proportion [36]. Furthermore, high-performing top adult athletes experience more knee flexion at the start [37]. Therefore, while the knee plays a crucial role in adult athletes, during the junior period, hip flexibility appears to contribute to the generation of greater power and is closely linked to short sprint performance.

The role of trunk flexibility was confirmed at a distance of 2.5–5 m. The intermediate range of 2.5–5 m showed no relationship with the other time intervals in either the simple correlation analysis or partial correlation analysis, controlling for age in months. This suggests that the 2.5–5 m range may have independent characteristics. In contrast, correlations were found between the 0–2.5 m and 5–10 m intervals. In these two intervals, the 0–2.5 m range showed a relationship with hip flexibility, while the 5–10 m range showed a relationship with lower-limb function in standing long jump, indicating a commonality in obtaining horizontal propulsive force. Previous studies on short sprints focused on lower-limb strength, flexibility, and kinematics [14,17,37]. This study focused on upper-body function and found a relationship between upper-body function and performance in the 2.5–5 m segment of short sprints. Therefore, to improve short sprint performance, which is strongly associated with lower-limb function, the importance of upper-body function and the need to address it as a whole-body movement have been suggested.

In the 5–10 m distance, the ability to generate horizontal propulsion appeared to contribute to faster times. This finding is strongly related to previous research which has shown that adult athletes have greater horizontal propulsion than junior athletes [38], and that greater horizontal force is associated with improved sprint performance [39]. The standing long jump measured in this study assessed the ability of athletes to generate horizontal propulsion, and those who achieved longer jumps demonstrated a superior ability to generate horizontal force. The 5–10 m distance represents the phase following the transition from a standing start to the running phase and is recognized as the beginning of the acceleration phase. Therefore, in line with previous findings, the ability to generate horizontal force may play a critical role in performance during this interval.

## 5. Limitations and Prospects

One limitation of this study is that step length could not be directly measured. Previous research has demonstrated that greater step length correlates with increased acceleration; however, this study did not explore the relationship between step length and other contributing factors. In future studies, a three-dimensional motion analysis could facilitate the precise measurement of step length and the kinematics of lower-limb movements, enabling a more comprehensive analysis. Furthermore, we plan to gather full-body biomechanics data to analyze the movement as a whole-body motion.

Another limitation is the small sample size. Furthermore, since the participants in this study were not elite young sprinters specializing in sprint training, it may not be possible to generalize the findings to all athletic levels or disciplines. Prior studies suggest that energy systems used in similar motions can vary depending on the athlete’s primary sport [40]. Therefore, in future studies, increasing the sample size and focusing on athletes who specialize in sprint-specific disciplines may yield additional insights.

In this study, we did not restrict participants’ dietary or supplement intake, which means that nutritional status was not accounted for as a factor potentially influencing performance. This presents a limitation in terms of the generalizability of our findings. Future research should consider tracking dietary habits and ergogenic supplement use to analyze their impact on performance in greater detail. Specifically, controlling for diet and supplements at least 48 h before testing would allow a more precise assessment of their effects on sprint and strength performance.

Moreover, implementing sprint training intervention programs across sports with varying characteristics could reveal which factors most effectively enhance sprint performance. For instance, by comparing the effects of sustained flexibility and strength training on sprint performance in sports with differing court sizes, such as basketball (smaller court) and soccer (larger field), we could identify which sprint distances benefit most from specific types of training. Such insights would contribute to more tailored and practical training programs specific to the demands of each sport.

## 6. Conclusions

Our results suggested that higher hip flexibility led to faster times in the 0–2.5 m, higher trunk flexibility led to faster times in the 2.5–5 m, and better standing long jump performance led to faster times in the 5–10 m interval. Even at a short distance of 10 m, this study implied that lower-body and upper-body flexibility were important for the 0–5 m distance, whereas muscle strength for propulsion became crucial beyond 5 m. The impact of hip flexibility was considered specific to junior athletes. Although sprinting is often thought of as a function of the lower limbs, core flexibility is also important, and it is necessary to train the whole body on a daily basis. An increase in body fat mass was suggested to slow down time across all distances. These results emphasize that even over a short distance such as 10 m, the factors influencing performance can change significantly. This highlights the importance of overall flexibility, propulsive power, and body fat regulation in junior short sprinters, as well as the need for daily training carefully tailored to the specific sprint distances required in each sport.

## Figures and Tables

**Table 1:** Correlation between sprint times in each section. The (mean value ± SD) value is indicated next to the variable name.

	0–2.5 m (*r*)	2.5–5 m (*r*)	5–10 m (*r*)
0–2.5 m (0.80 ± 0.07 s)	-	0.17	0.64 ***
2.5–5 m (0.53 ± 0.04 s)	0.17	-	0.24
5–10 m (0.89 ± 0.05 s)	0.64 ***	0.24	-
Age in month (136.46 ± 15.60 month)	−0.49 *	−0.47 *	−0.54 **
Height (145.73 ± 10.15 cm)	−0.23	−0.21	−0.45 *
Weight (37.72 ± 9.93 kg)	−0.02	0.06	−0.03
Fat-free mass (31.71 ± 6.48 kg)	−0.31	−0.17	−0.42 *
Body fat mass (6.01 ± 5.43)	0.34 †	0.31	0.45 *
Trunk rotation (right) (59.27 ± 9.96°)	0.04	−0.46 *	−0.08
Trunk rotation (left) (58.04 ± 10.62°)	−0.17	−0.53 **	−0.22
Hip flexion angle (right) (115.50 ± 10.00°)	0.07	0.27	0.30
Hip flexion angle (left) (117.27 ± 10.22°)	−0.25	0.13	−0.02
Isometric knee extension (87.23 ± 28.70 kg)	−0.26	−0.45 *	−0.32
Isometric knee flexion (39.58 ± 14.66 kg)	−0.48 *	−0.35 †	−0.60 *
Isotonic knee extension (40.81 ± 11.46 kg)	−0.28	−0.23	−0.34 †
Isotonic knee flexion (27.50 ± 7.56 kg)	−0.22	−0.37 †	−0.44 *
Repeated side-step (52.62 ± 5.90 times)	−0.37 †	−0.35 †	−0.39 *
Sit-ups (29.62 ± 4.68 times)	−0.37 †	−0.28	−0.39 †
Standing long jump (190.50 ± 16.78 cm)	−0.59 **	−0.41 *	−0.72 ***
Sit-and-reach (42.81 ± 6.58 cm)	−0.10	−0.33 †	−0.23
Whole-body reaction time (329.23 ± 48.12 ms)	0.29	0.17	0.40 *

† *p* < 0.1, * *p* < 0.05, ** *p* < 0.01, *** *p* < 0.001.

**Table 2:** Correlation between age in month and other factors.

	Age in Month (*r*)
0–2.5 m	−0.49 *
2.5–5 m	−0.47 *
5–10 m	−0.54 **
Height	0.79 ***
Weight	0.53 **
Fat-free mass	0.78 ***
Body fat mass	0.03
Trunk rotation (right)	0.39 *
Hip flexion angle (left)	0.44 *
Hip flexion angle (right)	−0.69 ***
Hip flexion angle (left)	−0.44 *
Isometric knee extension	0.69 ***
Isometric knee flexion	0.78 ***
Isotonic knee extension	0.63 ***
Isotonic knee flexion	0.74 ***
Repeated side-step	0.67 ***
Sit-ups	0.57 **
Standing long jump	0.78 ***
Sit-and-reach	0.44 *
Whole-body reaction time	−0.77 ***

* *p* < 0.05, ** *p* < 0.01, *** *p* < 0.001.

**Table 3:** Partial correlation coefficients with age in month controlled.

	0–2.5 m (*r*)	2.5–5 m (*r*)	5–10 m (*r*)
0–2.5 m	-	−0.08	0.52 ***
2.5–5 m	−0.08	-	−0.03
5–10 m	0.52 ***	−0.03	-
Height	0.29	0.30	−0.03
Weight	0.33	0.41 *	0.36 †
Fat-free mass	0.14	0.37 †	0.01
Body fat mass	0.40 *	0.37 †	0.55 **
Trunk rotation (right)	0.30	−0.34 †	0.17
Trunk rotation (left)	0.05	−0.40 *	0.03
Hip flexion angle (right)	−0.42 *	−0.09	−0.12
Hip flexion angle (left)	−0.60 **	−0.10	−0.35 †
Isometric knee extension	0.12	−0.19	0.09
Isometric knee flexion	−0.17	0.03	−0.34 †
Isotonic knee extension	0.03	0.09	0.00
Isotonic knee flexion	0.24	−0.03	−0.07
Repeated side-step	−0.07	−0.05	−0.04
Sit-ups	−0.13	−0.02	−0.11
Standing long jump	−0.39 †	−0.08	−0.56 **
Sit-and-reach	0.14	−0.16	0.02
Whole-body reaction time	−0.17	−0.34	−0.04

† *p* < 0.1, * *p* < 0.05, ** *p* < 0.01, *** *p* < 0.001.

## Data Availability

The original data presented in the study were uploaded to FigShare on 17 October 2024 and can be accessed via https://doi.org/10.6084/m9.figshare.27242148.v1.
